# Effects of Dietary Yeast Culture Supplementation on Growth Performance, Digestive Function, and Intestinal Health of Largemouth Bass *Micropterus salmoides*

**DOI:** 10.3390/microorganisms13071671

**Published:** 2025-07-16

**Authors:** Zheng Huang, Dingrui Mo, Xifeng Liu, Yuanfa He, Li Luo, Shimei Lin, Yongjun Chen

**Affiliations:** Key Laboratory of Freshwater Fish Reproduction and Development (Ministry of Education), Key Laboratory of Aquatic Science of Chongqing, College of Fisheries, Southwest University, Chongqing 400715, China; 18314022099@163.com (Z.H.); 18081717869@163.com (D.M.); 15178791211@163.com (X.L.); heyuanfa2022@swu.edu.cn (Y.H.); luoli1972@163.com (L.L.); linsm198@163.com (S.L.)

**Keywords:** yeast culture, *M. salmoides*, growth, digestive function, microbiota

## Abstract

This study was performed to investigate the effects of dietary yeast culture (YC) supplementation on growth performance, digestive function, intestinal inflammatory response, and microbiota composition of largemouth bass *Micropterus salmoides* (LMB). Six diets were formulated with graded levels of YC (0, 5, 10, 15, 20, and 30 g/kg), referred to as CON, YC5, YC10, YC15, YC20, and YC30, respectively. Each diet was assigned to four replicate tanks of LMB juveniles (initial body weight 8.11 ± 0.05 g) with twenty fish per tank. After an 8-week feeding trial, final body weight and specific growth rate showed an increasing trend with 5~20 g/kg YC and reached a maximum at 15 g/kg YC. Feeding ratio decreased, but feed efficiency ratio (FER) improved in response to dietary YC inclusion, and FER was higher in the YC10 fish than in the YC5, YC20, and YC30 fish. Proximate composition (moisture, protein, and lipid) of the whole fish was not affected by dietary YC levels. The activities of intestinal lipase and trypsin were higher in the YC10 fish, while the relative expression of interleukin-8 (*il-8*) and *il-1β* was downregulated in the hindgut of the YC15 fish compared with the CON fish. Histological examination showed that the villus height of the midgut, together with goblet cell density of the foregut and midgut, was higher in the YC10 and YC30 fish than in the CON fish. 16S rRNA sequencing showed that *Proteobacteria*, *Fusobacteria,* and *Firmicutes* dominated the intestinal microbiota in LMB. The decrease in harmful *Mycoplasma* accounted for the dramatic change in *Firmicutes* abundance, while the increase in *Cetobacterium* (specifically *C. somerae*) accounted for the change in *Fusobacteria* abundance in the gut of the YC10 and YC30 fish compared with the CON fish. The increase in the beneficial *Endozoicomonas* was the main reason for the change in Proteobacteria abundance in the intestine of the YC30 fish as compared with the CON fish. Taken together, the alteration of intestinal microbiota composition contributed to the improved digestive function and feed utilization in LMB fed YC-supplemented diets. Based on growth performance, the optimal YC level in the diet for LMB was 15 g/kg.

## 1. Introduction

Aquaculture, the fastest-growing food sector in the world, supplies nearly half of the total fish production for human consumption. China is the world’s largest aquaculture producer, accounting for about 60% of global aquaculture production [1]. As the aquaculture industry transitions from extensive farming to intensive farming, this sector faces persistently high stocking densities combined with rampant misuse of pharmaceuticals and antibiotics, resulting in elevated disease incidence throughout the production cycle [2,3]. This context highlights the importance of developing natural immunostimulants as functional feed additives to improve innate immunity and reduce disease susceptibility of fish. Yeast culture (YC), as a member of the symbiotic family, is a natural product of yeast fermentation, which contains abundant nutritional elements, such as amino acids, peptides, organic acids, and vitamins, as well as immune stimulants such as β-glucan, mannan oligosaccharides, and nucleotides [4,5,6]. It was previously reported that dietary β-glucan supplementation could improve both innate and adaptive immune responses [7,8,9], while liver health benefited from dietary mannan oligosaccharide or nucleotide inclusion in many fish species [10,11]. At present, YC has gained widespread attention in aquaculture with proven benefits. In addition to being used as a protein source to replace fish meal [6,12], YC can also be used as an immunomodulator to facilitate feed ingestion and digestion, enhance immunity and disease resistance, regulate gut microbiota, and boost antioxidant capacity, thereby improving the growth performance of aquatic animals [13,14,15,16,17]. It is noteworthy that the beneficial effects of dietary YC supplementation in aquatic animals are both dose-dependent and species-dependent [17,18].

Largemouth bass *Micropterus salmoides* (LMB) has emerged as a vital freshwater economic fish species in China, owing to its exceptional traits, including rapid growth, high nutritional value, and lack of intermuscular spines [19,20]. In China, there are two primary farming modes for LMB: pond culture and recirculating aquaculture. In pond culture systems, the typical stocking density ranges from 3.0 to 6.0 kg/m^3^, depending on management efficiency, while recirculating aquaculture systems can support densities up to 30 kg/m^3^ [21,22,23]. Generally, LMB fingerlings are introduced into water bodies for cultivation and must grow until they reach a weight of around 500 g to attain marketable size [24]. With breakthroughs in feed processing and extensive promotion of compound feed, both the farming areas and scale of LMB have rapidly expanded in recent years. In 2023, the total production of LMB in China exceeded 800,000 tons [25]. However, the application of compound feed is frequently associated with liver and intestine injuries in LMB [26,27], leading to disease outbreaks and enormous economic losses. To our knowledge, several studies have evaluated dietary yeast product supplementation on the growth performance and health status of LMB. For instance, Gong et al. reported that 15 or 30 g/kg dietary yeast hydrolysate improved antioxidant capacity and disease resistance of LMB without negative impact on growth performance [28]. Dietary YC inclusion at 30 g/kg attenuated the negative effects on growth performance, liver function, intestinal barrier, and microbiota of LMB fed high-starch or high-plant protein diets [15,29]. However, the optimal level of dietary YC has not yet been determined in LMB. Therefore, this study was performed to evaluate dietary YC levels on the growth performance, feed utilization, digestive function, and intestinal microbiota composition of LMB.

## 2. Materials and Methods

### 2.1. Experimental Design and Diets

YC was supplied by Chongqing Changnuo Biotechnology Co., Ltd. (Chongqing, China). The proximate composition and selective bioactive elements of YC are shown in Table 1. The formulation and proximate composition of the experimental diets are shown in Table 2. Six isonitrogenous (i.e., 53.7% protein) and iso-lipidic (i.e., 12.1%) diets were prepared with graded levels of YC (0, 5, 10, 15, 20, and 30 g/kg) at the expense of cellulose inclusion, designated as CON, YC5, YC10, YC15, YC20, and YC30, respectively. Fish meal, soy protein concentrate, and chicken meal were used as the main protein sources. Cassava starch was maintained at 10% as the carbohydrate source [18]. The feed ingredients were thoroughly pulverized and homogenously blended with water in the proportion of 40% by weight. Diet pellets were then processed through a twin-screw laboratory extruder (SG-YPYS-76, Xiamen, China) according to Chen et al. [30]. The experimental diets were air-dried, sealed in plastic bags, and stored in a −20 °C refrigerator.

### 2.2. Feeding Trial

LMB juveniles (Youlu No. 3 strain, about 4 g per fish) were purchased from Chongqing Three Gorges Ecological Fishery Co., Ltd. (Fuling, China). The fish were transported to our laboratory using a truck equipped with an insulated tank and an aeration system to maintain oxygenated water. Upon arrival, the fish were transferred into 2 circular plastic tanks (1500 L each) connected with a flow-through aquaculture system for 2 weeks of acclimatization. During that time, the fish were fed a commercial diet (Chongqing Haid Feed Co., Ltd., Chongqing, China) twice daily to apparent satiation. After the acclimatization, 480 LMB fingerlings (initial average weight, 8.11 ± 0.05 g) were selected and randomly distributed into 24 rectangular glass tanks (200 L each) operating as a recirculating system. Each diet was assigned to 4 replicate tanks of LMB with 20 fish per tank. During the next 8 weeks, the fish were fed twice a day (9:00 and 17:00) until apparent satiation, and feed intake was recorded accordingly.

The feeding trial was carried out under a controlled photoperiod (12 h: 12 h). During the experiment, the aquaculture system, including the filter materials (sponge and gravel) was cleaned once a week, while water quality parameters were monitored twice a week. Water quality indicators were maintained as follows: water temperature, 23.6 ± 1.8 °C; dissolved oxygen, 10.5 ± 0.4 mg/L, ammonia nitrogen, 0.10 ± 0.01 mg/L; nitrite, 0.12 ± 0.02 mg/L; pH, 7.40 ± 0.52.

### 2.3. Sample Collection

At the end of the feeding trial, the fish were fasted for 24 h before formal sampling. Twelve fish were randomly selected from each tank, and anesthetized with MS-222 (Sigma, Livonia, MI, USA) at a dose of about 75 mg/L. Three fish were used for the analysis of the proximate composition, including moisture, lipid, protein, and ash levels. Six fish were dissected for morphological measurement (including body weight, viscera weight, liver weight, and intraperitoneal fat weight). Then, gut samples of these fish were collected and immediately frozen in liquid nitrogen pending the analysis of digestive function indicators and microbiome composition. The remaining 3 fish were dissected to collect the intestine samples. Foregut and midgut were fixed in 4% paraformaldehyde solution for section preparation, while hindgut samples were immediately frozen in liquid nitrogen for real-time PCR analysis.

### 2.4. Proximate Composition Analysis

The proximate composition of the diets and whole fish was determined according to AOAC [31]. The moisture was measured by drying at 105 °C in an oven for 24 h until constant weight was achieved. The crude protein (calculated as N × 6.25) was analyzed using the Kjeldahl method. Crude lipid was quantified via Soxhlet extraction. The ash was determined by incinerating samples in a muffle furnace at 550 °C.

### 2.5. Analysis of Digestive Enzyme Activity

Intestine samples were homogenized with a tissue homogenizer in 9 volumes of phosphate-buffered saline (PBS). The homogenates were centrifuged at 3500× *g* for 10 min, and the supernatant was then collected for the analysis of digestive enzyme activities. The protein level of the supernatant was quantified by the biuret method. The activities of amylase (AMS, Cat. No. C061-1-1), lipase (LPS, Cat. No. A054-1-1), and trypsin (TPS, Cat. No. A080-2) were determined by spectrophotometric assays with commercial kits (Nanjing Jiancheng Institute of Biological Engineering, Nanjing, China). The activities of AMS and TPS were expressed as U/mg protein, while LPS activity was expressed as U/g protein.

### 2.6. Histological Examination

The intestine samples were fixed with 4% paraformaldehyde solution for at least 24 h and sent to Wuhan Servicebio Technology Co., Ltd. (Wuhan, China) for the preparation of paraffin sections. The samples were dehydrated in graded ethanol, embedded in paraffin, sliced into 5 μm thick sections, dewaxed, stained with hematoxylin-eosin (HE), and mounted with neutral resin. Then, the sections were observed and photographed using a microscope equipped with a computer image system (Olympus, DP73, Tokyo, Japan). The muscle layer thickness, the goblet cells count, and the villus height were quantified with the help of the ImageJ 23.0 software.

### 2.7. Identification of Target Genes

The complete cDNA sequence of target inflammatory genes, including interleukin-8 (*il-8*), *il-1β*, nuclear factor-κB (*nf-κb*), tumor necrosis factor-α (*tnf-α*), and transforming growth factor-β (*tgf-β*), as well as *β-actin,* was downloaded from the NCBI database (http://blast.ncbi.nlm.nih.gov/, accessed on 8 January 2025). Primer Premier 6.0 software was used for the primer design, and the specific primer sequences are shown in Table 3.

### 2.8. RNA Extraction, cDNA Synthesis, and Real-Time PCR

Total RNA was extracted from the intestine samples using RNAiso Plus reagent (TaKaRa, Kusatsu, Shiga, Japan). The RNA quality was verified through 1% agarose gel electrophoresis, and its concentration was quantified with an ultramicro spectrophotometer (Implen, Munich, Germany). Subsequently, total RNA was reverse transcribed into cDNA using the PrimeScript RT Master Mix Perfect Real-Time Kit (TaKaRa, Shiga, Japan). The cDNA products were stored at −20 °C pending real-time PCR analysis.

Real-time PCR analysis was performed using the fluorescent quantitative reagent SYBR^®^ Premix Ex Taq^TM^ II (TaKaRa, Shiga, Japan) on a Bio-Rad CFX96 real-time PCR system (Bio-Rad, Hercules, CA, USA), following the procedures described in our previous study [32]. *β-actin* was selected as the reference gene, and the relative expression of target genes was calculated using the formula (R = 2^−ΔΔCT^), as described by Livak and Schmittgen [33].

### 2.9. Intestinal Microbiome Composition

The gut samples of the CON, YC10, and YC30 fish (based on feed utilization) were sent to Beijing BioMarker Biotechnology Co., Ltd. (Beijing, China) for microbiome composition exploration through 16S rRNA sequencing, following the procedures described in our previous study [34]. Lima v1.7.0 and UCHIME v4.2 software were used to evaluate the quality of sequencing data. The sequences with similarity higher than 97% were defined as an operational taxonomic unit (OTU). The species classification information corresponding to the OTU was obtained by taxonomic annotation. The species composition and abundance were statistically analyzed at the level of phylum, genus, and species using QIIME 2. Alpha diversity indices (Chao1, ACE, Shannon, Simpson, and PD whole tree indices) were evaluated using mother vs. 1.30. Beta diversity was evaluated by principal component analysis (PCoA) based on unweighted UniFrac distance using QIIME.

### 2.10. Statistical Analyses

Data are expressed as mean ± SD (standard deviation) of four replicates. After normal distribution and variance homogeneity tests, the data were subject to one-way analysis of variance (ANOVA) and Duncan’s test. The significance level was set to 0.05. All the statistical analyses were performed using SPSS 23.0 software (SPSS Inc., Chicago, IL, USA).

## 3. Results

### 3.1. Growth Performance and Morphological Parameters

At the end of the feeding trial, the survival rate of all treatments was 100%. Data for growth performance, feed utilization, and biometric indicators are shown in Table 4. The final body weight and specific growth rate were markedly higher in the YC15 fish than in the YC30 fish (*p* < 0.05) but not significantly different from those in the other treatments (*p* > 0.05). Compared with the CON fish, the feed efficiency ratio (FER) was significantly increased in response to dietary YC inclusion (*p* < 0.05). Within the YC-supplemented treatments, FER was markedly higher in the YC10 fish than in the YC5, YC20, and YC30 fish (*p* < 0.05), but not significantly different from that of the YC15 fish (*p* > 0.05). The feeding ratio of all the YC-supplemented treatments was markedly lower than that of the CON fish (*p* < 0.05). All the tested biometric parameters, including viscerosomatic index, hepatosomatic index, and intraperitoneal fat ratio, were not affected by dietary YC inclusion (*p* > 0.05).

### 3.2. Proximate Composition of the Whole Fish

Data for the proximate composition of LMB are presented in Table 5. The moisture and protein content of LMB were not affected by dietary YC supplementation (*p* > 0.05). The lipid content was significantly higher in the YC5 fish than in the YC20 fish (*p* < 0.05), but not significantly different from that in the other treatments (*p* > 0.05). Compared with the CON fish, ash content was significantly increased in the YC30 fish but not in the other YC-supplemented treatments (*p* < 0.05).

### 3.3. Activity of Intestinal Digestive Enzyme

As shown in Table 6, amylase activity was significantly higher in the intestine of the YC15 fish than in that of the YC5 fish (*p* < 0.05), but not significantly different from the other treatments (*p* > 0.05). Intestinal lipase activity was markedly higher in the YC10 fish than in the other treatments (*p* < 0.05). Compared with the CON fish, trypsin activity was significantly increased in the intestine of the YC15 fish (*p* < 0.05) but not in the other YC-supplemented treatments.

### 3.4. Intestinal Histological Appearance

**Figure 1:**
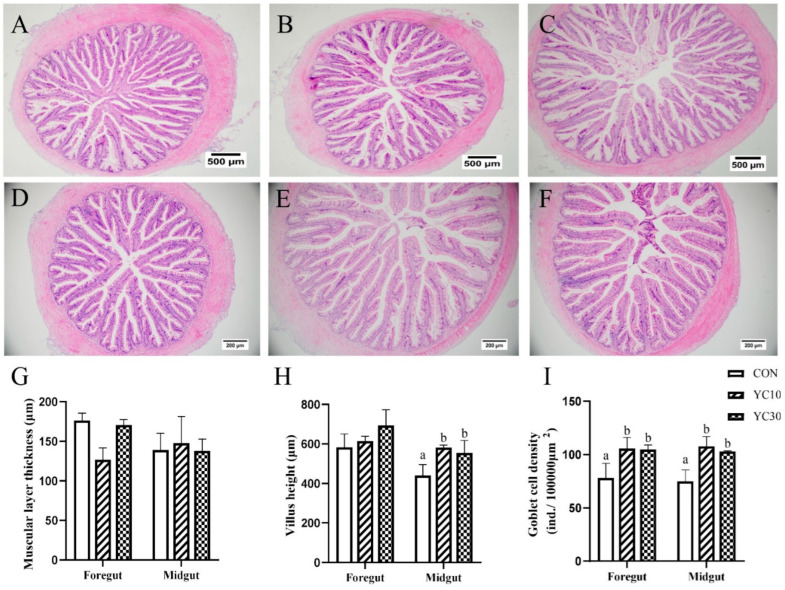
Effects of YC on intestinal morphology in LMB. (**A**–**C**) represent foregut sections from the CON, YC10, and YC30 groups, respectively; (**D**–**F**) represent midgut sections from the CON, YC10, and YC30 groups, respectively; (**G**), muscular layer thickness; (**H**), villus height; (**I**), goblet cell density. Data are presented as means ± SD (*n* = 4). Different letters on the error bar indicate significant differences among treatments (*p* < 0.05).

Data for the histological characteristics of the intestine are shown in Figure 1. Dietary YC inclusion at 15 or 30 g/kg did not affect the muscular layer thickness in the foregut and midgut of the LMB (*p* > 0.05). Dietary YC supplementation resulted in significantly increased villus height in the midgut (*p* < 0.05) but not in the foregut of LMB. Either 15 or 30 g/kg dietary YC inclusion markedly increased the density of goblet cells in the foregut and midgut of LMB (*p* < 0.05).

### 3.5. The Expression of Key Genes Involved with Inflammatory Response in Hindgut

**Figure 2:**
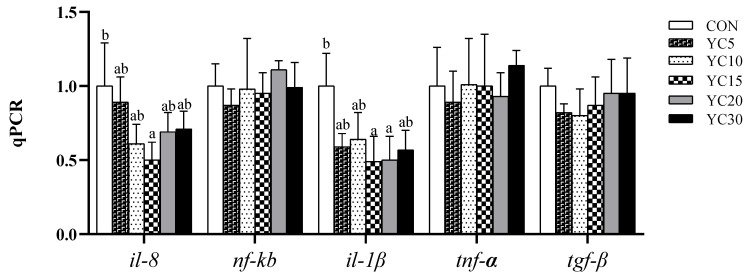
Relative expression of representative genes involved in the inflammatory response in the hindgut of LMB. Data are presented as means ± SD (*n* = 4). Different letters on the error bar indicate significant differences among treatments (*p* < 0.05).

As shown in Figure 2, the mRNA level of *il-8* in the hindgut was markedly lower in the YC15 fish than in the CON fish (*p* < 0.05), but not significantly different from the other treatments (*p* > 0.05). Compared with the CON fish, the relative expression of *il-1β* was significantly downregulated in the hindgut of the YC10 and YC15 fish (*p* < 0.05). The mRNA levels of *nf-κb*, *tnf-α,* and *tgf-β* in the hindgut of LMB were not affected by dietary YC inclusion (*p* > 0.05).

### 3.6. Intestinal Microbiota Composition

**Figure 3:**
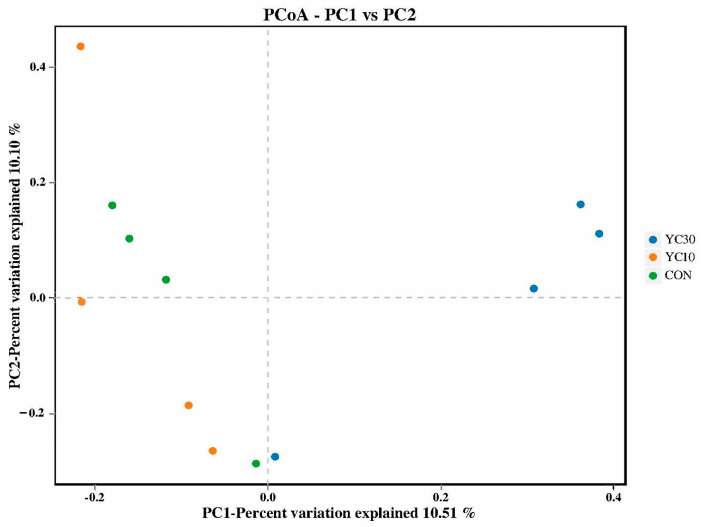
Beta diversity of the intestinal microbiota by principal coordinates analysis.

The sequencing coverage of the intestinal microbiome exceeded 99.9% in all treatments (Table 7). Among the alpha diversity indicators, Chao 1, Shannon, and Simpson indices in the intestinal microbiota were significantly higher in the YC10 and YC30 fish than in the CON fish (*p* < 0.05). The PD whole tree was significantly higher in the YC10 fish than in the CON fish (*p* < 0.05), but not significantly different from that of the YC30 fish (*p* > 0.05). The principal coordinates analysis (PCoA) showed that most samples in the same treatment were close to each other (Figure 3).

**Figure 4:**
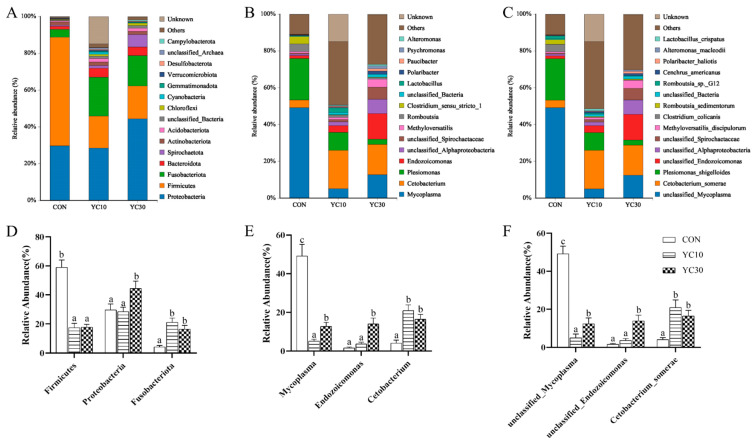
Effects of dietary YC inclusion on the intestinal microbiota composition of LMB. (**A**,**D**) phylum level; (**B**,**E**) genus level; (**C**,**F**) species level. Data are presented as means ± SD (*n* = 4). Different letters on the error bar indicate significant differences among treatments (*p* < 0.05).

Data for the intestinal microbiota composition of LMB are shown in Figure 4. At the phylum level, the dominant bacteria in the intestine of LMB were *Firmicutes*, *Proteobacteria,* and *Fusobacteriota* (Figure 4A). With the increase in dietary YC inclusion from 0 to 30 g/kg, the abundance of *Proteobacteria* and *Fusobacteria* in the intestine of LMB was significantly increased (*p* < 0.05), while the opposite was true for the abundance of *Firmicutes* (Figure 4D). At the genus level, the dominant bacteria were *Mycoplasma*, *Endozoicomonas,* and *Cetobacterium* (Figure 4B). The abundance of *Mycoplasma* in the intestinal microbiota was significantly higher in the CON fish than in the YC10 and YC30 fish (*p* < 0.05), while the opposite was true for the abundance of *Cetobacterium* (Figure 4E). The abundance of *Endozoicomonas* in the intestinal microbiota was markedly higher in the YC30 fish than in the other treatments (*p* < 0.05). At the species level, *unclassified Mycoplasma*, *unclassified Endozoicomonas,* and *C. somerae* were the dominant bacteria (Figure 4C). These bacteria changed in response to dietary YC supplementation in the same way as the genera to which they belonged (Figure 4F).

## 4. Discussion

Throughout the entire experimental period, the stocking density (0.81~7.32 kg/m^3^) was maintained much lower than the upper limit of the recirculating aquaculture system in China (30.0 kg/m^3^) to avoid negatively affecting the growth performance and health status of LMB. At the end of the 8-week feeding trial, growth performance (specifically final body weight and specific growth rate) exhibited no statistically significant differences between the YC-supplemented and CON fish. However, it demonstrated a progressive elevation with increasing dietary YC concentrations, culminating in maximal values at the 15 g/kg supplementation level, beyond which a decline was evident. Growth performance data indicate that supplementing 15 g/kg YC in the diet for LMB offers substantial practical benefits in aquaculture. At an incremental cost of CNY 30 per metric ton compared to the YC-free feed, this formulation increases LMB production by approximately 79.3 kg per ton, generating additional revenue of CNY 2537. Feng et al. reported that the feeding ratio was not responsive to dietary YC inclusion at 10 or 30 g/kg in LMB fed a high-starch diet [29]. In the present study, however, the YC-supplemented treatments obtained a lower feeding ratio than the CON fish. This indicated that the improvement in growth performance in LMB in response to dietary YC inclusion benefited from a higher feed efficiency ratio. Among the YC-supplemented treatments, the YC10 fish exhibited he best feed efficiency ratio, which was higher than that of the YC5, YC20, and YC30 fish. In line with our study, the improvement of growth performance and/or feed utilization in response to dietary YC inclusion was observed in the same fish species [29] and many other fish species [13,17,18,35]. In this experiment, the viscerosomatic index and hepatosomatic index of LMB were not affected by dietary YC inclusion, which was similar to the results in gibel carp *Carassius auratus* [12] and Ussuri catfish *Pseudobagrus ussuriensis* [17].

Digestive enzymes are crucial when fish use a large amount of ingested nutrients for rapid growth and development [36,37]. The activity of digestive enzymes is indicative of the physiological status of fish and the quality of functional feed additives [38,39]. In this study, although amylase activity remained unaffected by dietary YC supplementation, intestinal lipase and trypsin activities of LMB were improved with 10 g/kg YC compared with those of the CON fish. The enhancement of digestive function was consistent with the feed efficiency ratio, which peaked in the YC10 fish. Notably, in hybrid grouper *Epinephelus fuscoguttatus*^♀^ × *E. lanceolatus*^♂^, intestinal amylase, lipase, and trypsin activities were enhanced with either 20 or 40 g/kg dietary YC supplementation [18], suggesting species-specific responses to dietary YC administration.

Goblet cells play a crucial role in maintaining intestinal homeostasis and immune regulation through mucus secretion and bicarbonate provision in the intestine [40,41]. Villus height serves as a key physiological indicator of nutrient absorption capacity in animals [42]. Based on growth performance and feed efficiency ratio, intestine samples from the CON, YC10, and YC30 fish underwent further histological evaluation. HE staining revealed that the density of goblet cells in both foregut and midgut of the YC-supplemented treatments was higher than that of the CON fish. Furthermore, the YC10 and YC30 fish demonstrated higher villus height in the midgut than the CON fish. Interleukin-8 (*il-8*) and *il-1β* are pro-inflammatory cytokines that activate immune cells, induce cytokine release, and stimulate local/systemic inflammatory responses [43,44,45]. In the present study, the mRNA level of *il-8* was lower in the hindgut of the YC10 fish, while the relative expression of *il-1β* was downregulated in the hindgut of the YC10 and YC15 fish compared with the CON fish. These results suggested that dietary YC supplementation at 10~15 g/kg enhanced intestinal homeostasis and nutrient absorption, ultimately improving feed utilization and growth performance of LMB. In line with our results, dietary YC inclusion improved intestinal barriers and attenuated inflammatory response in the same species [28] and other fish species [4,46].

Intestinal microbiota plays a vital role in host nutrition, physiology, and immune processes [47]. Within this ecosystem, both pathogenic and beneficial bacteria coexist [48]. Beneficial probiotics sustain microbial homeostasis while enhancing host nutrition through the synthesis of microbial-derived metabolites exceeding host requirements. This metabolic activity improves nutrient bioavailability and partitioning [49,50]. In the present study, 16S rRNA sequencing revealed the beneficial roles of microbiota in the intestinal health of LMB fed with 10 and 30 g/kg YC. Compared with the CON fish, PD whole tree of the intestinal microflora was increased in the YC10 fish, while the Chao 1, Shannon and Simpson indices were increased in both the YC10 and YC30 fish, suggesting that dietary YC inclusion increased both bacterial richness and diversity in LMB intestine, which is similar to the results of Zhou et al. [51]. *Proteobacteria*, *Fusobacteria,* and *Firmicutes* dominated the microbiota in the LMB intestine in this study. The relative abundance of *Mycoplasma* increases when the host is suffering from metabolic disorders or diseases [52,53]. In this experiment, the decrease in *Mycoplasma* drove the dramatic change in *Firmicutes* abundance, while the increase in *Cetobacterium* (specifically *C. somerae*) abundance explained *Fusobacteria* changes in the gut microbiota of the YC-supplemented fish compared with the CON fish. *C. somerae* abundance is positively correlated with the production of acetic acid, propionic acid, and vitamin B12 in the body [54]. *Endozoicomonas* could not only act as the center of protein transformation and production that could be beneficial for the fish [55] but also play a role in the host health or disease, such as competitive exclusion of pathogenic bacteria [56,57]. In this study, the abundance of *Endozoicomonas* was increased in the YC30 fish compared with the CON fish, which was the main reason for the Proteobacteria changes. These findings demonstrate that dietary YC supplementation not only enhances nutrient production/absorption via metabolite synthesis but also inhibits detrimental microbiota proliferation, ultimately improving digestive efficiency and growth performance in LMB.

## 5. Conclusions

Under the present experimental conditions, the optimal YC level in the diet for LMB was 15 g/kg based on growth performance. YC demonstrates tremendous application potential in LMB feed, delivering improved feed utilization, enhanced growth performance, healthier intestines, and contributions to more sustainable aquaculture practices at relatively low costs. However, long-term feeding trials are required to validate the consistent beneficial effects of dietary YC on the growth performance and health status of LMB under practical farming conditions.

## Figures and Tables

**Table 1:** The nutrient composition of YC (dry matter basis).

Composition	Content
Crude protein (%)	22.8
Crude fiber (%)	12.5
Crude ash (%)	9.5
Moisture (%)	8.1
Yeast counting (×10^8^/g)	8.3
β-glucan (%)	3.7
Mannan (%)	3.1
Nucleic acid/nucleotide (%)	2.8

**Table 2:** The formulation and proximate composition of the experimental diets.

Ingredients (%)	CON	YC5	YC10	YC15	YC20	YC30
Fish meal ^1^	34.0	34.0	34.0	34.0	34.0	34.0
Chicken meal ^1^	15.0	15.0	15.0	15.0	15.0	15.0
Soy protein concentrate ^1^	18.0	18.0	18.0	18.0	18.0	18.0
Wheat gluten ^1^	5.00	5.00	5.00	5.00	5.00	5.00
Cassava starch ^1^	10.0	10.0	10.0	10.0	10.0	10.0
Fish oil ^2^	2.00	2.00	2.00	2.00	2.00	2.00
Soybean oil	4.00	4.00	4.00	4.00	4.00	4.00
Vitamin C phosphate ^1^	0.20	0.20	0.20	0.20	0.20	0.20
Methionine ^1^	0.15	0.15	0.15	0.15	0.15	0.15
Monocalcium phosphate ^1^	2.00	2.00	2.00	2.00	2.00	2.00
Vitamin and mineral premix ^3^	2.00	2.00	2.00	2.00	2.00	2.00
Choline chloride ^1^	0.50	0.50	0.50	0.50	0.50	0.50
Bentonite ^1^	3.00	3.00	3.00	3.00	3.00	3.00
Microcrystalline cellulose	4.15	3.65	3.15	2.65	2.15	1.15
Yeast culture ^4^	0	0.50	1.00	1.50	2.00	3.00
Analyzed nutrient composition (% dry matter)						
Moisture	3.64	3.52	3.11	3.93	3.32	3.58
Crude protein	53.6	53.7	53.8	54.0	54.0	53.3
Crude lipid	11.6	12.3	12.2	12.1	12.3	12.4
Crude ash	14.5	14.2	14.1	14.1	14.1	14.2

^1^ Supplied by Chongqing Sitejia Biotechnology Co., Ltd. (Chongqing, China). ^2^ Supplied by Chongqing HAID Feed Co., Ltd. (Chongqing, China). ^3^ Supplied by Guangzhou Chengyi Aquatech Co., Ltd. (Guangzhou, China). ^4^ Supplied by Chongqing Changnuo Biotechnology Co., Ltd. (Chongqing, China).

**Table 3:** Nucleotide sequences of the primers for real-time PCR analysis.

Target Genes	Forward Primer (5′–3′)	Reverse Primer (3′–5′)	Product Size (bp)	Accession No.
*nf-κb*	CCACACCCCCTACCATCTCA	ACTCCTCGTCCTCCTCTTCCTC	198	XM_038698033
*tnf-α*	AAATAGTGATTCCTCAAGACGG	TGAACAGTATGGCTCAGATGG	126	XM_038723994
*il-1β*	CGTGCCAACAGTGTGAAGAC	TGGACAGAACAACGGGACTAC	193	XM_038733429
*il-8*	TCCTGGCTGCTCTGGCTCTC	GGATGGCCCTCCTGTTAATGG	111	XM_038704089
*tgf-β*	GCTCAAAGAGAGCGAGGATG	TCCTCTACCATTCGCAATCC	118	XM_038693206
*β-actin*	TTCACCACCACAGCCGAAAG	TCTGGGCAACGGAACCTCT	179	XM_038738148

*nf-κb*, nuclear factor-κB; *tnf-α*, tumor necrosis factor-α; *il-1β*, interleukin-1β; *il-8*, interleukin-8; *tgf-β*, transforming growth factor-β.

**Table 4:** Growth performance and morphological parameters of LMB fed different diets.

Diets	CON	YC5	YC10	YC15	YC20	YC30
IBW (g)	8.14 ± 0.20	8.19 ± 0.34	8.07 ± 0.24	8.16 ± 0.34	8.15 ± 0.18	8.23 ± 0.13
FBW (g)	68.4 ± 0.5 ^ab^	68.8 ± 4.3 ^ab^	71.3 ± 0.9 ^ab^	73.2 ± 1.0 ^b^	69.2 ± 1.0 ^ab^	67.0 ± 2.4 ^a^
SGR (%) ^1^	3.78 ± 0.01 ^ab^	3.81 ± 0.16 ^ab^	3.84 ± 0.03 ^ab^	3.93 ± 0.08 ^b^	3.85 ± 0.02 ^ab^	3.68 ± 0.06 ^a^
FER ^2^	0.93 ± 0.00 ^a^	0.96 ± 0.00 ^b^	1.00 ± 0.01 ^c^	0.98 ± 0.01 ^bc^	0.97 ± 0.01 ^b^	0.97 ± 0.01 ^b^
FR (%) ^3^	3.03 ± 0.00 ^b^	2.95 ± 0.06 ^a^	2.88 ± 0.05 ^a^	2.94 ± 0.01 ^a^	2.93 ± 0.02 ^a^	2.90 ± 0.03 ^a^
VSI (%) ^4^	8.69 ± 0.13	8.42 ± 0.27	8.41 ± 0.16	8.69 ± 0.17	8.67 ± 0.19	8.61 ± 0.24
HSI (%) ^5^	1.72 ± 0.10	1.78 ± 0.06	1.69 ± 0.08	1.71 ± 0.08	1.68 ± 0.08	1.60 ± 0.04
IPF (%) ^6^	3.10 ± 0.22	2.80 ± 0.39	2.83 ± 0.34	2.98 ± 0.17	2.93 ± 0.04	2.99 ± 0.13

Data are presented as means ± SD (*n* = 4). Means in the same row with different superscripts indicate significant differences (*p* < 0.05). IBW, initial body weight. FBW, final body weight. ^1^ Specific growth rate = 100 × ln (final body weight/initial body weight)/days of feeding. ^2^ Feed efficiency ratio = (final body weight − initial body weight)/feed intake. ^3^ Feeding ratio (%) = 100 × feed intake/[(initial body weight + final body weight)/2]/days of feeding. ^4^ Viscerosomatic index (%) = 100 × viscera weight/body weight. ^5^ Hepatosomatic index (%) = 100 × liver weight/body weight. ^6^ Intraperitoneal fat ratio IPF (%) = 100 × intraperitoneal fat weight/body weight.

**Table 5:** The proximate composition in the whole body of LMB (% wet weight).

Diets	CON	YC5	YC10	YC15	YC20	YC30
Moisture	68.8 ± 0.6	67.9 ± 0.4	68.3 ± 0.8	68.1 ± 0.6	68.6 ± 0.4	68.2 ± 0.2
Protein	18.4 ± 0.1	18.1 ± 0.4	18.4 ± 0.2	18.1 ± 0.1	18.5 ± 0.4	18.5 ± 0.4
Lipid	10.5 ± 0.9 ^ab^	11.3 ± 0.4 ^b^	10.8 ± 0.5 ^ab^	10.4 ± 0.3 ^ab^	9.81 ± 0.34 ^a^	10.5 ± 0.5 ^ab^
Ash	3.55 ± 0.17 ^a^	3.78 ± 0.16 ^ab^	3.81 ± 0.07 ^ab^	3.80 ± 0.03 ^ab^	3.58 ± 0.09 ^a^	3.92 ± 0.07 ^b^

Data are presented as means ± SD (*n* = 4). Means in the same row with different superscripts indicate significant differences (*p* < 0.05).

**Table 6:** Activities of digestive enzymes in the intestine of LMB.

Diets	CON	YC5	YC10	YC15	YC20	YC30
AMS	0.046 ± 0.01 ^ab^	0.040 ± 0.01 ^a^	0.049 ± 0.01 ^ab^	0.057 ± 0.02 ^b^	0.043 ± 0.01 ^ab^	0.048 ± 0.01 ^ab^
LPS	34.0 ± 7.4 ^a^	33.1 ± 4.9 ^a^	44.5 ± 8.3 ^b^	32.9 ± 3.4 ^a^	31.7 ± 6.3 ^a^	29.0 ± 4.9 ^a^
TPS	25.3 ± 5.6 ^a^	33.9 ± 1.6 ^ab^	36.6 ± 9.6 ^b^	33.1 ± 9.4 ^ab^	32.6 ± 6.4 ^ab^	32.8 ± 9.2 ^ab^

Data are presented as means ± SD (*n* = 4). Means in the same row with different superscripts indicate significant differences (*p* < 0.05). Amylase (AMS, U/mg prot); lipase (LPS, U/g prot); trypsin (TPS, U/mg prot).

**Table 7:** Effects of dietary YC inclusion on alpha diversity of intestinal microbiota in LMB.

Diets	Chao 1	PD Whole Tree	Shannon	Simpson	Goods Coverage
CON	185 ± 37 ^a^	7.26 ± 2.89 ^a^	2.39 ± 0.29 ^a^	0.71 ± 0.22 ^a^	1.0000
YC10	241 ± 47 ^b^	21.7 ± 1.4 ^b^	6.34 ± 0.11 ^b^	0.85 ± 0.14 ^b^	0.9999
YC30	226 ± 11 ^b^	18.4 ± 1.9 ^ab^	6.27 ± 0.49 ^b^	0.84 ± 0.16 ^b^	1.0000

Data are presented as means ± SD (*n* = 4). Means in the same row with different superscripts indicate significant differences (*p* < 0.05).

## Data Availability

The original contributions presented in this study are included in the article. Further inquiries can be directed to the corresponding author.
